# Coronary computed tomography angiography with 320-row detector and using the AIDR-3D: initial experience

**DOI:** 10.1590/S1679-45082013000300025

**Published:** 2013

**Authors:** Roberto Sasdelli, Cesar Higa Nomura, Ana Carolina Sandoval Macedo, Danilo Perussi Bianco, Fernando Uliana Kay, Gilberto Szarf, Gustavo Borges da Silva Teles, Hamilton Shoji, Pedro Vieira Santana Netto, Rodrigo Bastos Duarte Passos, Rodrigo Caruso Chate, Walther Yoshiharu Ishikawa, João Paulo Bacellar Costa Lima, Marcelo Assis Rocha, Vinícius Neves Marcos, Bruna Bonaventura Failla, Marcelo Buarque de Gusmão Funari

**Affiliations:** 1 Hospital Israelita Albert Einstein, São Paulo, SP, Brazil; 2 Universidade Metodista de São Paulo, São Bernardo do Campo, SP, Brazil

**Keywords:** Coronary angiography, Coronary artery disease, Multidetector computed tomography, Radiation, ionizing, Exposure control to radiation, Image processing, computed-assisted, Myocardial ischemia, Diagnostic imaging, Cardiac-gated imaging techniques, Cardiac imaging techniques

## Abstract

Coronary computed tomography angiography (coronary CTA) is a powerful non-invasive imaging method to evaluate coronary artery disease. Nowadays, coronary CTA estimated effective radiation dose can be dramatically reduced using state-of-the-art scanners, such as 320-row detector CT (320-CT), without changing coronary CTA diagnostic accuracy. To optimize and further reduce the radiation dose, new iterative reconstruction algorithms were released recently by several CT manufacturers, and now they are used routinely in coronary CTA. This paper presents our first experience using coronary CTA with 320-CT and the Adaptive Iterative Dose Reduction 3D (AIDR-3D). In addition, we describe the current indications for coronary CTA in our practice as well as the acquisition standard protocols and protocols related to CT application for radiation dose reduction. In conclusion, coronary CTA radiation dose can be dramatically reduced following the “as low as reasonable achievable” principle by combination of exam indication and well-documented technics for radiation dose reduction, such as beta blockers, low-kV, and also the newest iterative dose reduction software as AIDR-3D.

## RESUMO

A angiotomografia computadorizada de coronárias (angioTC de coronárias) é um excelente método de imagem não invasivo para avaliar a doença arterial coronariana. Atualmente, a dose de radiação efetiva estimada da angioTC de coronárias pode ser reduzida em tomógrafos de última geração com múltiplos detectores, como o tomógrafo com 320 fileiras de detectores (320-CT), sem prejuízo na acurácia diagnóstica da angioTC de coronárias. Para reduzir ainda mais a dose de radiação, novos algoritmos de reconstrução iterativa foram recentemente introduzidos por vários fabricantes de tomógrafos, que atualmente são utilizados rotineiramente nesse exame. Neste trabalho, apresentamos nossa experiência inicial na angioTC de coronárias utilizando o 320-CT e o *Adaptive Iterative Dose Reduction 3D* (AIDR-3D). Apresentamos ainda as indicações mais comuns desse exame na rotina da instituição bem como os protocolos de aquisição da, angioTC de coronárias com as atualizações relacionadas a essa nova técnica para reduzir a dose de radiação. Concluímos que a dose de radiação da angioTC de coronárias pode ser reduzida seguindo o princípio *as low as reasonable achievable* (tão baixo quanto razoavelmente exequível), combinando a indicação de exame com técnicas bem documentadas para a diminuição da dose de radiação, como o uso de betabloqueadores e a redução do kV, com os mais recentes aplicativos de reconstrução iterativa para redução da dose de radiação, como o AIDR-3D.

## INTRODUCTION

Coronary computed tomography angiography (CTA) examination's role was established on the last American College of Cardiology/American Heart Association (ACC/AHA) guidelines as a non-invasive imaging method to evaluate coronary artery disease and some cardiovascular diseases^(1)^. Nowadays, coronary CTA estimated effective radiation dose can be dramatically reduced by using state-of-theart scanners, such as dual-source CT (DSCT) and 320-row detector CT (320-CT)^(2)^, without changing coronary CTA diagnostic accuracy^(3)^. To optimize and further reduce the radiation dose, new iterative reconstructions algorithms were recently released by several scanners manufacturers^(4)^, and now they are routinely used in coronary CTA. This paper presents our first experience in using coronary CTA with 320-CT and the Adaptive Iterative Dose Reduction 3D (AIDR-3D). In addition, it describes the coronary CTA indications as well as the acquisition protocols related to this new CT application for radiation dose reduction.

## THE CORONARY CTA INDICATIONS

Each coronary CTA indication demands a proper CTA scan protocol, which might increase the radiation dose. For example, protocols designed to evaluate coronary artery bypass graft surgery and “triple-rule-out coronary CTA” often require higher doses than standard coronary CTA^(1)^.

The majority of patients referred to our institution for coronary CTA have prior equivocal cardiovascular exams, such as the treadmill stress test and the singlephoton emission computed tomography (SPECT), as main exam indication.

Other indications for coronary CTA related to current appropriate criteria score (ACCF/SCCT/ACR/AHA/ASE/ASNC/NASCI/SCAI/SCMR2010 Appropriate Use Criteria for Cardiac Computed Tomography) are displayed on chart 1 ^(1)^.

**Chart 1:** Indications for coronary computed tomographic angiography

Indication	Clinical features	Score*
Non-acute symptomatic patients	Interpretable electrocardiogram AND able to exercise	A-7**
	Uninterpretable electrocardiogram OR unable to exercise	A-8
Acute (urgent presentation) symptomatic patients	Normal electrocardiogram and cardiac biomarkers	A-7
	Non-diagnostic electrocardiogram OR equivocal cardiac biomarkers	A-7
	Acute chest pain of uncertain cause	U-6***
Use of computed tomographic angiography in the setting of prior test results – prior electrocardiogram exercise testing	Normal electrocardiogram exercise test and continued symptoms	A-7
	Prior electrocardiogram exercise testing and Duke Treadmill Score - intermediate risk findings	A-7
	Discordant electrocardiogram exercise and imaging results	A-8
	Evaluation of computed tomographic calcium score >100 in symptomatic or between 100-400	A-8
Risk assessment post-revascularization (percutaneous coronary intervention or coronary artery bypass grafting surgery)	Coronary artery bypass grafting surgery evaluation in symptomatic patients (ischemic equivalent)	A-8
	Asymptomatic; prior left main coronary stent with stent diameter ≥3mm	A-8
Evaluation of cardiac structure	Assessment of anomalies of coronary arterial and other thoracic arteriovenous vessels	A-9
	Assessment of complex adult congenital heart disease	A-8
	Evaluation of ventricular morphology and systolic function	A-7
Evaluation of cardiac structure and function – evaluation of intra and extracardiac structures	Characterization of native or prosthetic cardiac valves (suspected dysfunction)	A-8
	Evaluation of cardiac mass (suspected tumor or thrombus)	A-8
Preoperative assessment	Prior biventricular pacemaker placement	A-8
	Prior cardiac surgery to assess coronary	A-8

Adapted from: ACCF/SCCT/ACR/AHA/ASE/ASNC/NASCI/SCAI/SCMR2010 Appropriate Use Criteria for Cardiac Computed Tomography^(1)^.

* Score 7 to 9: test is generally acceptable and is reasonable approach for the indication; score 4 to 6: test may be generally acceptable and may be a reasonable approach for the indication; score 1 to 3: test is not generally acceptable and is not a reasonable approach for the indication.

** A: appropriate test for specific indication;

*** U: uncertain test for specific indication.

## PATIENT PREPARATION TO CORONARY CTA

Patients referred to coronary CTA can receive oral or intravenous beta blockers to reduce heart rate unless they have contraindications, such as overt heart failure, asthma, or atrioventricular conduction abnormalities^(5)^. Beta blocker dose protocols of our institution are detailed on chart 2.

**Chart 2:** Institutional lowering heart rate protocols with orally and intravenously beta blockers

HR (beats/minutes)	Beta blocker dose
<55	None
55<HR<60	5mg IV of metoprolol tartrate (Seloken^®^), if necessary
60<HR<70	5-15 mg IV of metoprolol tartrate (Seloken^®^) 15 minutes before scan
70<HR<80	40mg of propranolol hydrochloride (Inderal^®^) orally or metoprolol tartrate (Seloken^®^) 5-15 mg IV, 15-45 minutes before scan
80<HR<90	100mg of metoprolol tartrate (Seloken^®^) orally or 40mg of propranolol hydrochloride (Inderal^®^) orally at least 1 hour before the scan
HR>90	100mg of metoprolol tartrate (Seloken^®^) orally at least 1 hour before the scan

IV: intravenous; HR: heart rate.

Sublingual isosorbide dinitrate (3.75mg) is administrated routinely prior to coronary CTA if not contraindicated due to pulmonary hypertension, severe aortic stenosis, the use of phosphodiesterase type 5 inhibitors (such as the use of sildenafil citrate in the last 24 hours, or tadalafil in the last 72 hours) and migraine^(5)^.

## THE SCANNER

In our institution, coronary CTAs are performed in two 320-row CT scanners (Aquilion ONE, Toshiba Medical Systems, Tochigi-ken, Japan). All patients are scanned with prospective electrocardiographic (ECG) gating/triggering, independently of heart rate. This technique uses forward-looking prediction of R-wave timing, step-and-shoot non-spiral acquisition with no table motion during imaging, and unique cone beam reconstruction^(6)^.

The scanning plan is based on body mass index (BMI), in order to apply the lowest kV and mA for each patient (Chart 3), using the Sure Exposure 3D^®^ (Tochigi-ken, Japan) with an automatic exposure control system^(7)^.

**Chart 3:** 320-row computed tomographic scanner acquisition parameters for three different coronary computed tomographic angiography protocols

Exam	Detectors/collimation/tube rotation speed	Kilovoltage (kV)	Current intensity (mA)	FOV (mm)	Range (mm)	Pitch	Wide Volume	Filter	RR interval phases	Dose modulation software	CM injection
Prospective coronay computed tomographic	320/0,5/0,35	80-135	Sure Exposure 3D CTA Standard	(M) 220	120	0	Off	Cardiac Stent (AIDR-Standard)	70, 75 and 80%	Prosp CTA (Sure Cardio)	Flow: 4,5-5mL/s CM volume: 75mL
Coronary computed tomographic/revascularized pacient	320/0,5/0,35	80-135	Sure Exposure 3D CTA Standard	(M) 220	232	0	On	Cardiac Stent (AIDR-Standard)	70, 75 and 80%	Prosp CTA (Sure Cardio)	Flow: 4,5-5mL/s CM volume: 100mL
Aortic prothesis	80/0,5/0,35	100 kV when BMI <30	1^st^ sequence: Sure Exposure 3D CTA Standard	(M) 220	—	1^st^ sequence: standard	Off	Body Standard and Lung	Angiographic: coronary, thoracic and abdominal aorta until iliac bifurcation	CTA/CFA continous (Sure Cardio)	Flow: 4,5-5mL/s CM volume is equal to 10 units plus the acquisition time
		120 kV when BMI >30	2^nd^ sequence: Sure Exposure Low Dose			2^nd^ sequence*: fast					

* Variable helical pitch allows division in acquisition parameters. Thoracic acquisition is electrocardiogram-triggered. Abdominal acquisition is performed without electrocardiogram trigger, which is automatically turned off allowing a single apnea examination and lowering contrast administration.

Sure Exposure 3D^®^, SureCardio^®^ and AIDR-Standard^®^ are trade registered marks and data was supplied by Toshiba Medical Systems (Tochigi-ken, Japan).

FOV: field of view; CM: contrast media; BMI: body mass index; CTA: coronary computed tomography angiography; CFA: cardiac function assessment.

The z-axis coverage or the range varies from 10 to 16cm, and the 12 to 14cm range is used in about 75% of our patients. Scanner standard values provided by the manufacturer are displayed on chart 3. There is no table movement, so pitch is zero. Reconstruction algorithm uses the “half” protocol, which increases temporal resolution to 175ms^(8)^, and Xact + (on), that corrects cone beam angle.

Iodinated contrast media (Henetix^®^ 350mg/mL, Guerbet, Lille, France) is injected using dual-head injection system, volume ranges from 50 to 100mL, according to patient's BMI and coronary CTA indication, followed by a 50mL flush of saline solution^(5)^.

## DOSE REDUCTION STRATEGY: ITERATIVE RECONSTRUCTION

CT scan images are formed from reconstructions of projections of the radiation detected in multiple angles in a tomography scan, such as back-projection (BP) or filtered back-projection (FBP) associated to iterative reconstructions, since 1970^(4)^. The term “iterative” refers to a method of successive approximations until satisfactory agreement with an arbitrary starting image. Therefore, iterative reconstructions by definition repeat the reconstruction process several times, and are much slower than analytic methods^(4)^.

The increase of low-dose CT scanning implies reduction of the number of photons reaching the detector, and results on a decrease in the signal-to-noise ratio and more strike artifacts^(8,9)^.

**Figure 1:**
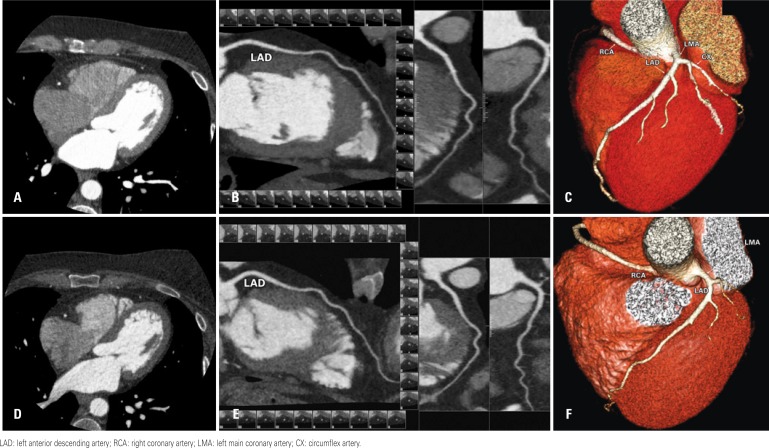
Comparison between two coronary computed tomographic angiography examinations of the same patient, the first without AIDR-3D (Figures A-C) and the second with AIDR-3D (Figures D-F). The noise is different on axial computed tomographic images (Figures A and D), but the curved-MIP (Figures B and E) and 3D volume-rendering (Figures C and F) reconstructions are similar. Estimated effective radiation dose from coronary computed tomography angiography only and full examination were respectively 6.6mSv and 8.8mSv (exam without AIDR-3D), and 1.97mSv and 3.9mSv (exam with AIDR-3D)

**Figure 2:**
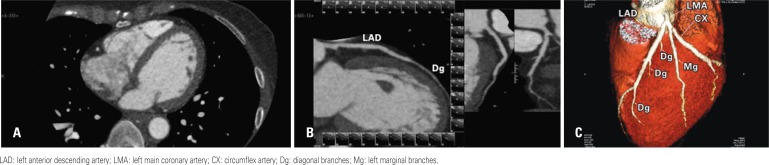
Coronary computed tomographic angiography examination with AIDR-3D. Axial image (A), curved-MIP (B) and 3D volume-rendering (C) reconstructions. Estimated effective radiation dose from coronary computed tomographic angiography only and full examination were 0.43mSv and 1.02mSv, respectively

The AIDR-3D is a recent iterative reconstruction algorithm composed by lots of operations launched by Toshiba Medical Systems (Tochigi-ken*,* Japan). The aim of the operations in the projection data space is to reduce streak artifacts caused by photon starvation. Therefore, a 3D-smoothing filter is applied to the photon count values, which performance is fu rther enhanced with statistical models of the noise and the scanner. In the meantime, the AIDR-3D operations occurs in the image reconstruction domain, in order to obtain iterative noise reduction^(8)^. The final process involves a weighted blending of the iterative and the primary reconstruction to create AIDR-3D image. As a result of this blending, the images retain a more typical CT appearance, as if they were simply acquired with standard CT exposure parameters^(10)^ (Figure 1). Nowadays, the AIDR-3D can be applied to all acquisition modes for routine clinical use and is able to eliminate up to 50% of image noise, resulting in dose reduction of up to 65%^(8)^ (Figure 2).

## CONCLUSION

In conclusion, coronary computed tomography angiography radiation dose can be dramatically reduced, following the ALARA (“as low as reasonable achievable”) principle, combining the exam indication with well-documented technics in coronary computed tomography angiography, such as beta blockers, low-kV, and the use of dose reduction software, as AIDR-3D.
